# Interview with Duncan Campbell: After the Draft Sequence, What Next for the Human Genome Mapping Project Resource Centre?

**DOI:** 10.1002/cfg.83

**Published:** 2001-06


**Comparative and Functional Genomics**
*Comp Funct Genom* 2001; **2**: 176–179.
**DOI:** 10.1002/cfg.83

Interview: Duncan Campbell

### After the draft sequence, what next for the Human Genome Mapping Project Resource Centre?

Dr R. Duncan Campbell **is the Director of the Medical Research Council (MRC) funded UK Human Genome Mapping Project Resource Centre (HGMP-RC), on the Wellcome Trust Genome Campus at Hinxton, near Cambridge, UK. The Centre provides both biological and bioinformatics resources to the UK genomics community, and also has research groups supporting these activities and investigating separate projects.**

***CFG: The HGMP-RC is broadly arranged into three Divisions: Bioinformatics, Biology Services and Research. Let's tackle these one at a time. The draft sequence release will no doubt have encouraged many researchers to get on-line and use bioinformatics tools to mine the dataset, can you tell me about the projects that the Bioinformatics team are working on to facilitate such investigations?***

RDC: With the huge amount of draft sequence now available, we have seen increased use of the bioinformatics tools already on offer from the HGMP-RC, such as NIX and PIX. NIX is a suite of programs for database screening by BLAST, repeat masking and exon prediction for the identification of genes from genomic sequences. PIX is a protein equivalent of NIX, with BLAST searches, programs for domain and sequence motif detection, prediction of secondary structure and other tools.

We are currently working on tools for microarray bioinformatics. Microarrays are clearly a very important research tool since they allow monitoring of the expression levels of large numbers of genes in parallel. We are developing tools, which will hopefully become available shortly, to support the use of the microarray service which we have set up. In addition to providing improved tools for data acquisition we will offer a public database to facilitate mining of the microarray data. We are also continuing to develop EMBOSS, a suite of tools for DNA and protein sequence analysis, which will expand the functions offered by the Genetics Computer Group (GCG) package. We are currently redeveloping our website in order to make it bigger, better and more interactive for our users, and our hope is that we will have a new website which will go live in the next few months. The site will include access and ordering for the existing services and information and support for new services as they come on-line.

***CFG: The Biology Services Division supplies a continuously expanding range of clone libraries, and hosts a Linkage Hotel, which enables groups to send a lab member to the HGMP-RC to perform genetic screens for their loci of interest. Are there any new plans for this Division in the light of the genome draft? Will the Linkage Hotel continue to be available; do you see a shift away from genetic screens, or are there still plenty of loci out there waiting to be pinned down?***

RDC: There certainly are plenty of disease loci still waiting to be pinned down, but what I think will change is how the screening will be done. So far we have used microsatellites in our genotyping, but we are now shifting towards using Single Nucleotide Polymorphisms (SNPs). That is not to say that we will stop using microsatellites, they have proved to be a very useful tool for genetic linkage studies. In

Copyright © 2001 John Wiley & Sons, Ltd.

fact, we actually hope to expand our capacity in this area, from the current 0.5 million genotypes, up to 1–1.5 million microsatellite genotypes. We are currently looking very seriously at SNP analysis, since SNPs offer a lot of advantages over microsatellites, partly because of their increased density, but also because they can alter gene expression if they are located in the UTRs, and can alter gene function if they are located in the coding sequence. SNP analysis will provide a tremendous amount of useful information, but at the moment we need to select a platform for SNP genotyping. A range of options are available, including Mass Spectrometry, fluorescent capillary sequencing, Invader and Taqman approaches and we are validating these alternatives to decide which one we will use. We are aiming to combine high-throughput (that is to say the ability to do tens of thousands of SNP genotypes a day) with the best cost, to allow us to provide a service similar to what we offer for microsatellite screening.

Another key aspect of Biology Services in the past has been supplying clones such as BACs and cDNAs. We are currently expanding our range of full-length cDNA resources, and are distributing human and mouse cDNA clones as part of the Mammalian Gene Collection (Strausberg *et al.*, (1999). We are also supplying resources that are important for functional genomics; for example, another addition is a *C. elegans* RNAi resource made by Julie Ahringer's laboratory in Cambridge (UK) for suppression of the expression of each gene across *C. elegans* chromosome I (Fraser *et al.*, (2000). We are also making microarrays by PCR amplification of cDNA inserts and spotting onto glass slides. The PCR products are from human and mouse cDNA libraries and will be made available through Biology Services.

The activities which Biology Services are involved in are continually expanding; in order to cope with this expansion, the Division will be moving to larger premises at the Babraham Campus. We have also obtained additional funding from the MRC for further posts to support the increased demand for resources and genotyping. Our main aim (which can be quite a daunting task in the fast moving field of functional genomics) is to keep pace with the new types of services and resources needed by the research community.

***CFG: Many of the interests of the Research Division stem from your long-standing interest in the Major Histocompatibility Complex (MHC) class III region. Perhaps you could give our readers a brief backgrounder on this interesting region of the human genome?***

RDC: The MHC spans about 4 Mb of DNA and contains around 224 loci, at least 128 of which are predicted to be expressed (MHC Sequencing Consortium, (1999). The class III region is just over 800 kb long and contains 61 loci, it is the most gene dense region in the human genome. The MHC region is of interest, not only because of the large number of genes involved in immune and inflammatory responses, but also because many human autoimmune diseases show genetic susceptibility mapping to this region. One of the key things we want to do is identify which of the genes are involved in these diseases. One way to do this is to look for SNPs in the genes and then genotype them in DNA from patients to see if they have a role in the disease. The SNPs located within genes and their control regions are the best candidates for these studies as they are most likely to affect gene function. A crucial part of this research is to have access to DNA from large patient populations, and we already have important collaborations, for example with John Todd, who has a large collection of diabetes patient samples and Dominic Kwiatkowski who has samples we can use to assess the effects of MHC SNPs on malaria susceptibility. We would like to expand the range of diseases we are screening our SNPs against and we are keen to set up new collaborations that would enable us to look at diseases such as RA and multiple sclerosis.

***CFG: There are several functional genomics research projects underway at the HGMP-RC using genes from the MHC class III region as models for mechanisms that might be genome wide, can you tell us a little about these projects?***

RDC: One of the great things about genes in the class III region is that they illustrate all of the same features as genes from around the genome that one might want to investigate. The region is very gene dense; the genes are very close together, with some overlapping at their 5′ ends and some at their 3′ ends, so there are going to be some very interesting aspects of the regulation of these genes. There is a large amount of alternative splicing of the class III genes. At least 40% of genes analysed undergo alternative splicing, with a wide range of complexity, from having two or three splice variants, all the way

Copyright © 2001 John Wiley & Sons, Ltd.

up to 21, in the case of one particular gene. In most of those genes that we have studied, the splice variation affects the amino acid sequence and therefore may well affect the function of the gene products.

Class III gene products exhibit a wide range of subcellular localisations, from the cytoplasm, to the nucleus, to the cell surface. We are using and developing a range of techniques to look at the functions of these proteins; for the cell surface proteins we are looking at the ligands that they bind and the intracellular pathways that they activate.

We are also using yeast 2-Hybrid screens and developing them into a high throughput mode to identify the interacting partners of the intracellular proteins. Finally, we are systematically tagging each of the encoded proteins to define their subcellular location. This approach will facilitate affinity purification of expressed proteins and interacting partners.

***CFG: The research division also has groups working on other genomes: the mouse sequencing group and the* Fugu *genomics group. The mouse sequencing group has joined in the project to sequence the mouse genome, can you tell me more about that? Do you have any plans to exploit their data?***

RDC: The mouse sequencing group is acting as part of the MRC funded UK Mouse Sequencing Programme, which includes the Sanger Centre, the MRC Mammalian Genetics Unit and UK Mouse Genome Centre at Harwell, the MRC Human Genetics Unit (HGU) in Edinburgh and Imperial College in London. The consortium is focussed on generating finished sequence for four regions of the mouse genome, on chromosomes 2, 4, 13 and X, which underpin work at Harwell and HGU. The HGMP-RC will generate 25 Mb of finished sequence and the Sanger centre will produce a further 25 Mb of sequence. Harwell and HGU supply mapped clones to the HGMP-RC, and the sequence data generated here is annotated by the ENSEMBL group at the European Bioinformatics Institute (EBI). Like the human genome data, it is also deposited in the public databases to become part of the larger international effort. Harwell and HGU will then use this data to identify genes that cause the phenotypes they have already been studying in their mouse models.

***CFG: Sequencing* Fugu *cosmids was initially suggested (and quite widely taken up) as a faster alternative for gene identification than sequencing human clones. I expect that the availability of the draft sequence will have changed the emphasis of the* Fugu *Genomics group; can you tell us what role you now see it playing?***

RDC: The Joint Genome Institute (JGI) of the US Department of Energy, the Institute for Systems Biology (in Seattle, USA), Greg Elgar's group here at the HGMP-RC and the Institute for Molecular and Cell Biology (in Singapore) have formed a consortium to generate a draft sequence of the *Fugu* genome. That will be very important because it will allow a three-way comparison between human, mouse and *Fugu* genomic DNA. In the last year, Greg's team have already shown that these types of comparisons allow easier identification not only of coding sequences, but can also reveal enhancers and regulatory sequences in the promoter regions and introns of genes. In some cases, there is a stronger contrast in the sequence conservation with *Fugu*, which helps to highlight these regions better than the human and mouse comparison alone.

In addition to generating the *Fugu* genome sequence, the group is also involved in the development of new software tools to facilitate the identification of regulatory regions. They are repackaging and developing existing sequence comparison tools as well as the new tools, for analysis and visualisation of these three way comparisons.

***CFG: You have mentioned a microarray group, can you give us more detail on what services you hope to provide to the community and which technologies are you considering offering?***

RDC: The ability to use microarrays to look at the expression of tens of thousands of genes is one of the key technologies in functional genomics. Through Tom Freeman we have set up a microarray group here, which is producing human and mouse microarrays on glass slides, loaded with PCR amplified products from cDNA clones. One of our aims is to provide the PCR products from the cDNA clones, in addition to the microarrays themselves, to the community, for use in their own work. We also want to provide informatics support for this service, including tools for data analysis and encouraging deposition of the data into a database and providing opportunities for data mining. This is a fast moving field and another aspect of the remit of the group is to keep up with the current technologies. In addition to supplying the glass

Copyright © 2001 John Wiley & Sons, Ltd.

slide arrays to the community, we also have an Affymetrix GeneChip system and provide access to this via a 'Microarray Hotel', run in a similar way to the Linkage Hotel system. We will work with those groups who choose to use the arrays, they will get advice and support on aspects such as preparing their RNA samples (to the high quality which is crucial for obtaining meaningful results), and we will supply protocols and other support information with the slides and through our website.

***CFG: An important part of your role is keeping up to date with new techniques and approaches for functional genomics, so I'm sure there are other new projects in the pipeline that you would like to tell us about, are you looking at proteomics for example?***

RDC: Research projects for the future include expanding our capabilities in proteomics, including developing tools for *in silico* predictions of protein function and protein-protein interactions and continuing to develop our high throughput yeast 2-Hybrid systems for detection of interacting protein pairs. We will also be doing 1D and 2D-gel analysis of whole cell protein extracts and further immunoprecipitations of tagged protein complexes, these will be followed by Mass Spectrometric identification of the proteins. We are also looking at novel developments in proteomics such as phage-antibody based arrays and protein arrays to see how these can be incorporated into the research work we are doing and also how these might be supplied, or provided as services, to the community in the future.

### Related websites

UK HGMP-RC
**http://www.hgmp.mrc.ac.uk/**

Mammalian Gene Collection
**http://mgc.nci.nih.gov/**

Todd lab webpage – Diabetes Genetics
**http://diesel.cimr.cam.ac.uk/todd/**

Kwiatkowski lab webpage – Malaria Genetics
**http://www.well.ox.ac.uk/malaria/malaria_genetics.htm**

UK Mouse Sequencing Programme
**http://mrcseq.har.mrc.ac.uk/**

The Sanger Centre
**http://www.sanger.ac.uk/**

MRC Mammalian Genetics Unit and UK Mouse Genome Centre
**http://www.har.mrc.ac.uk/**

MRC Human Genetics Unit
**http://www.hgu.mrc.ac.uk/**

Imperial College of Science, Technology and Medicine
**http://www.ic.ac.uk/templates/front_index_3.asp?P=178**

The European Bioinformatics Institute (EBI)
**http://www.ebi.ac.uk**

The Joint Genome Institute (JGI)
**http://jgi.doe.gov/tempweb/**

The Institute for Systems Biology
**http://www.systemsbiology.org/**

Institute for Molecular and Cell Biology
**http://www.imcb.nus.edu.sg/index.shtml**

### References

Fraser AG, Kamath RS, Zipperlen P, Martinez-Campos M, Sohrmann M, Ahringer J. 2000. Functional genomic analysis of *C. elegans* chromosome I by systematic RNA interference. *Nature* **408**: 325–330.

MHC Sequencing Consortium. 1999. Complete sequence and gene map of a human Major Histocompatibility Complex. *Nature* **401**: 921–923.

Strausberg RL, Feingold EA, Klausner RD, Collins FS. 1999. The mammalian gene collection. *Science* **286**: 455–457.

---

The Interview articles of *Comparative and Functional Genomics* aim to present a commentary on topical issues in genomics studies. They are a personal critical analysis, by the interviewee, of current work in their field of expertise, and aim at providing implications for future genomics studies. This interview was undertaken by the Managing Editor, Dr Joanne Wixon.

**Dr R. Duncan Campbell**
Director, MRC UK HGMP-RC, Hinxton, Cambridge, CB10 1SB, UK

Copyright © 2001 John Wiley & Sons, Ltd. 
